# Ship navigation behavior pattern mining: Representations and intelligent methods

**DOI:** 10.1016/j.isci.2026.117550

**Published:** 2026-09-25

**Authors:** Yu Xiong, Da Wang, Luo Chen

**Affiliations:** 1 College of Electronic Science and Technology, National University of Defense Technology, Changsha 410073, China

**Keywords:** ship navigation behavior pattern, behavior pattern mining, behavior pattern representations, deep-representation learning, large-model-enhanced methods

## Abstract

Maritime transportation is a core pillar of global trade. Mining meaningful behavioral patterns from ship trajectories is essential for characterizing ship movement regularities, supporting maritime traffic management, and improving maritime situational awareness. Although substantial progress has been made in anomaly detection, trajectory prediction, and scenario-specific behavior recognition, publicly available labeled datasets remain limited by incomplete labeling schemes, and ship behavior modeling still lacks a unified framework. To clarify these gaps, this review systematically examines data sources, labeled datasets, behavior modeling, mining methods, and practical applications related to ship navigation behavior pattern mining. It further proposes a three-layer behavior modeling framework that organizes ship behavior into physical behavior, interactive behavior, and semantic behavior. This framework connects low-level motion states, spatiotemporal interactions with the maritime environment and other ships, and high-level semantic interpretation, thereby providing a structured basis for understanding behavioral patterns in ship trajectories. Building on this review, we summarize the major challenges and future research directions in the field. This study provides a reference for subsequent research and aims to promote the development of ship navigation behavior pattern mining, particularly by extending spatial data mining methods for static data toward spatiotemporal pattern mining of dynamic streaming data from moving objects.

## Teaser

Navigation system; Applied sciences

## Introduction

Maritime transportation has become a core component of global trade because of its large carrying capacity, low cost, and suitability for long-distance transport. According to the United Nations Conference on Trade and Development,^1^ more than 80% of global trade by volume is carried by sea, and global demand for maritime transportation is expected to continue increasing in the coming decades. As shipping traffic expands, maritime traffic flows are becoming denser, navigational environments are becoming more complex, and operational tasks are becoming more diverse. These changes make ship navigation behavior more dynamic in maneuvering, more interactive with the surrounding environment, and richer in behavioral patterns.^2^ Abnormal ship behavior, as well as conflicts between a ship and other ships or environmental constraints, may increase navigational safety risks. Therefore, cognitive modeling and intelligent mining of ship navigation behavior are important foundations for ensuring maritime traffic safety, maintaining navigational order, and supporting intelligent maritime surveillance and regulation.

Ship navigation behavior refers to a continuous spatiotemporal process in which ship operators complete tasks such as fishing, transportation, and special operations within navigable areas, including terminals, channels, and straits, while interacting with fishing grounds, ports, islands, and other environments of interest.^3^ Accordingly, ship navigation behavior patterns can be understood as spatiotemporal events with specific semantics that emerge from the coupling between ship trajectory sequences and the distribution of geographic environments. This definition suggests that ship behavior is not merely a sequence of motion states, but also a process shaped by navigation tasks, geographic context, environmental interaction, and semantic interpretation.

Trajectory-based behavioral patterns have been widely applied across diverse fields, including road traffic accident analysis, electric-vehicle charging configuration optimization, behavior-based camera-motion optimization, port ship-speed forecasting, and low-carbon shipping pathway evaluation.^4^^,^^5^^,^^6^^,^^7^^,^^8^

Existing reviews have examined AIS data processing and governance,^9^^,^^10^^,^^11^ maritime anomaly detection,^12^^,^^13^^,^^14^^,^^15^ trajectory prediction,^16^^,^^17^^,^^18^ and vessel-behavior analysis and modeling.^19^ These studies provide substantial coverage of individual analytical tasks, model families, and operational applications. The remaining gap is therefore not the absence of another algorithmic catalog, but the lack of clear boundaries among analytical objects that are frequently grouped under “ship behavior”. Context-free motion patterns, vessel-object relations, context-conditioned event meanings, regional traffic aggregates, and downstream risk estimates represent different levels and types of evidence, yet they are often discussed within the same hierarchy. This conflation makes terminology inconsistent and prevents meaningful comparison among methods whose outputs are not conceptually equivalent. A related problem is the failure to distinguish representational abstraction from aggregation scale: single-vessel maneuvers, pairwise interactions, and regional traffic structures differ primarily in the number and organization of the entities represented, rather than necessarily forming successive semantic levels.

In this Review, ship navigation behavior denotes a temporally bounded vessel-activity process evidenced by vessel-motion evolution and, where relevant, by relations to other vessels, geographic objects, operational contexts, or maritime rules. Kinematic patterns describe regularities derived from vessel motion without requiring an external reference, whereas interaction patterns describe the temporal evolution of vessel-vessel or vessel-environment relations. Trajectory patterns are geometric or spatiotemporal regularities that do not automatically carry behavioral meaning, and traffic patterns are regional aggregates of vessel distributions, flows, routes, or interaction structures. A maneuver is an observable change or sustained configuration of motion; an event is a bounded, context-conditioned interpretation involving identifiable participants, time, and location; and intention is a latent task or goal hypothesis that cannot be inferred from trajectory geometry alone. An anomaly is a deviation assessment relative to a specified statistical, contextual, operational, or regulatory reference, rather than a primitive behavior category.

The core literature considered here includes studies that derive kinematic primitives, relational patterns, behavior segments, event hypotheses, intentions, rule-related interpretations, or anomaly assessments from temporally linked vessel observations. Trajectory-prediction studies are included only when their representation, interaction, uncertainty, or validation mechanisms directly contribute to behavior mining. Traffic-flow analysis is treated as regional or collective context, whereas collision and accident risks are downstream inferences based on behavior evidence and independent risk models. Emission estimation, route optimization without behavior interpretation, static attribute classification, and cross-domain methods without a direct representational, inferential, evaluative, or verification role are outside the core scope. On this basis, the Review asks how ship-behavior evidence should be defined and organized, how representational abstraction can be separated from aggregation scale, and how methodological design choices affect representation, supervision, uncertainty, and verification. It contributes a scale-aware spatiotemporal semantic framework, a multi-axis synthesis of non-mutually exclusive methodological paradigms, an assessment of behavior-annotated datasets, and a clear separation between behavior representation and downstream risk or decision processes. The framework is conceptual and comparative rather than a formal ontology or an empirically validated end-to-end architecture. The overall review structure is shown in Figure 1.

**Figure 1:**
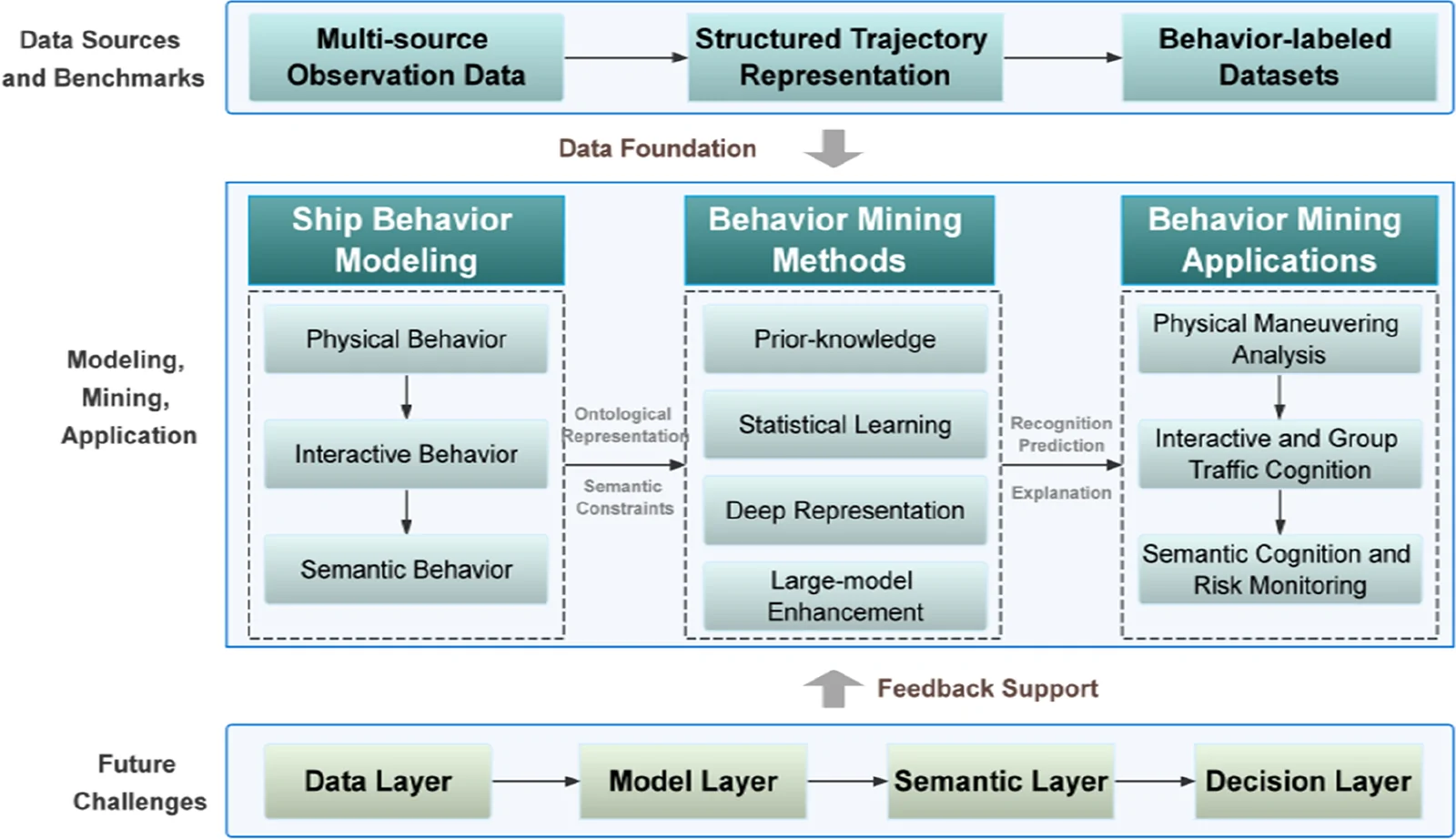
Research framework for mining ship behavior patterns

To improve transparency, this review used a structured literature-identification procedure rather than a formal systematic-review protocol. Core searches were conducted in the Web of Science Core Collection, Scopus, IEEE Xplore, and CNKI for literature available up to June 30, 2026. ScienceDirect and Google Scholar were used for supplementary retrieval, together with institutional repositories, official maritime data and standards websites, and backward and forward citation tracking. Search terms combined AIS, ship or vessel trajectories, and maritime traffic with behavior, maneuvering, interaction, intention, anomaly, semantic modeling, ontology, knowledge graphs, machine learning, deep learning, generative models, and large language models. English- and Chinese-language journal articles and full conference papers were considered. Studies were included when temporally linked vessel observations were substantively used for behavior representation, recognition, segmentation, interaction modeling, semantic interpretation, explanation, evaluation, or verification. Studies focused exclusively on preprocessing, attribute classification, static traffic analysis, prediction, optimization, emissions, or geometric risk calculation were excluded unless a specific representation, interaction, uncertainty, or validation mechanism directly contributed to behavior mining.

## Data sources and datasets for ship behavior pattern mining

With the widespread application of Earth observation, mobile communication, and Internet of Things technologies, cooperative reporting systems and non-cooperative sensing systems have enabled near-real-time monitoring of vessel attributes and positions.^9^^,^^20^ Cooperative systems, including AIS, the Vessel Monitoring System (VMS), and Long-Range Identification and Tracking (LRIT), rely on shipborne terminals to report identity, attributes, and position. Non-cooperative systems, including radar, optical remote sensing, and sonar, observe vessels without relying on onboard self-reporting. Depending on platform coverage and acquisition frequency, non-cooperative observations may provide detections, local tracks, or repeated position estimates, whereas cooperative reporting systems can generate identity-linked spatiotemporal sequences over longer periods. These sequences constitute the principal data source for the behavior-representation tasks reviewed here.^10^ Accordingly, this section focuses on cooperative reporting data, establishes a common trajectory-state notation, and reviews annotated vessel-behavior datasets.

### Cooperative reporting systems and trajectory representation

Among cooperative reporting systems, AIS is a global shipping data exchange system promoted as mandatory by the International Maritime Organization (IMO), and it provides relatively high data dimensionality and fine spatiotemporal resolution.^9^^,^^10^ VMS is designed primarily for fisheries monitoring and management and is usually characterized by medium-frequency updates and compressed dynamic parameters. LRIT supports the global identification and tracking of ships for shipping safety, maritime security, and marine environment protection, but it is characterized by very low-frequency sampling and limited dynamic attributes.

Because of its broad coverage, strong continuity, and rich attribute information, AIS is commonly used as the main data source for ship navigation behavior pattern mining.^10^ According to the International Telecommunication Union Radiocommunication Sector (ITU-R) M.1371 standard,^21^ AIS data include static information, dynamic information, voyage-related information, and safety and auxiliary information, as summarized in Table 1.

**Table 1:** AIS broadcast message data

Type	Content	Update frequency	Example
Static attributes (S)	MMSI, IMO number, call sign and ship name, length and beam, ship type, and so forth.	usually every 6 min, or when data are modified or requested	MMSI = 413123456; IMO = 9876543; ship_name = OCEAN STAR; ship_type = Cargo; length = 180 m; beam = 28 m
Dynamic attributes (D)	spatial position, UTC, SOG, COG, HDG, navigational status, ROT, and so forth.	usually from 2 s to 3 min, dynamically adjusted according to speed, course variation, and equipment type	time = 2024–06–01 10:30:00; lon = 121.5200; lat = 31.2300; sog = 12.5 kn; cog = 86.4°; heading = 87°; rot = 0.2°/min; nav_status = Under way using engine
Voyage-related messages (V)	draught, hazardous cargo type, destination, ETA, and so forth.	usually every 6 min, or when data are modified or requested	draught = 9.8 m; destination = Shanghai; eta = 2024–06–02 08:00; cargo_type = Category B
Safety and auxiliary messages (M)	safety-related text messages, binary application messages, interrogation and response messages, base-station UTC and date responses, channel management, and so forth.	sent on demand	safety_text = Restricted area ahead; interrogation_target_mmsi = 413123456; requested_message = 5; channel = AIS1/AIS2

Note: AIS, automatic identification system; MMSI, Maritime Mobile Service Identity; IMO, International Maritime Organization; UTC, Coordinated Universal Time; SOG, speed over ground; COG, course over ground; HDG, heading; ROT, rate of turn; ETA, estimated time of arrival.

Regardless of the data source, a ship navigation trajectory is represented as a time-ordered sequence of spatiotemporal observations. For a target vessel *v*, let the time associated with the *i*-th dynamic observation be

$$\begin{array}{cc} t_{i}^{\left(v\right)}=\text{time}\left(D_{i}^{\left(v\right)}\right)\text{,} & t_{1}^{\left(v\right)}<t_{2}^{\left(v\right)}<\cdots <t_{n_{v}}^{\left(v\right)}\text{.} \end{array}$$

The corresponding AIS trajectory and its *i*-th observation are represented as

$$\begin{array}{cc} X^{\left(v\right)}={\left(p_{i}^{\left(v\right)}\right)}_{i=1}^{n_{v}}\text{,} & p_{i}^{\left(v\right)}=\left(S^{\left(v\right)},D_{i}^{\left(v\right)},V_{i}^{\left(v\right)}\right)\text{.} \end{array}$$

Here, *n*_*v*_ is the number of trajectory observations, and *i* is the observation index. *S*^(*v*)^, $D_{i}^{\left(v\right)}$, and $V_{i}^{\left(v\right)}$ denote the static-attribute vector, the dynamic-attribute vector observed at $t_{i}^{\left(v\right)}$, and the voyage-related information temporally associated with that observation, respectively. Because AIS fields are updated asynchronously, complete voyage-related information is not assumed to be available at every sampling time; the temporal-association and missing-value rules must be stated by the corresponding application.

The geographic position extracted from the dynamic attributes is denoted by

$$l_{i}^{\left(v\right)}=\text{loc}\left(D_{i}^{\left(v\right)}\right)=\left(\lambda_{i}^{\left(v\right)},\varphi_{i}^{\left(v\right)}\right)\text{,}$$

where $\lambda_{i}^{\left(v\right)}$ and $\varphi_{i}^{\left(v\right)}$ are longitude and latitude in angular units. These geographic coordinates are not used directly in Euclidean distance calculations.

Compared with the relatively complete spatiotemporal attributes provided by AIS, VMS and LRIT are limited by lower sampling frequencies and missing attribute fields.^22^^,^^23^ VMS sampling intervals can extend to 30 min or even 2 h, and motion attributes such as HDG and ROT are often not retained. LRIT is typically sampled every 6 h and retains only identity information and two-dimensional coordinates, making it difficult to support fine-grained and time-sensitive behavior analysis.

### Annotated datasets for ship behavior patterns

Annotated datasets for ship behavior are an important foundation for connecting the key components of ship navigation behavior pattern mining and overcoming existing technical bottlenecks. Compared with the rapid growth of methodological studies, however, systematic organization of annotated ship behavior datasets remains insufficient. To address this issue, this review examines journal articles and open-source databases and systematically summarizes trajectory-based annotated datasets and research samples for ship behavior patterns. Because nearly all currently public, reusable, and representative behavior annotation datasets are constructed from AIS data, whereas VMS and LRIT data are constrained by regulation, privacy, and legal requirements and are therefore difficult to develop into standardized open benchmarks, this review focuses on public AIS datasets and a small number of accessible research samples. To support meaningful cross-dataset comparison, Table 2 separates dataset scale and coverage from annotation unit, semantic granularity, label provenance, and public availability.

**Table 2:** Comparative characteristics of public AIS-based behavior datasets and accessible research samples

Dataset	Scale	Spatiotemporal coverage	Annotation unit, label granularity, and behaviors	Label provenance and public availability
HawaiiCoast_GT^24^	88,749,176 AIS points; 2,622 vessels; 208 labeled tracks; 154 linked incidents	Hawaii coastal waters; Jan. 2017–Dec. 2020	incident-linked track level; 19 event/incident classes, including collision, grounding, smuggling, search and rescue, and law enforcement; incident coverage non-exhaustive	manual AIS-incident report correlation; public on Zenodo; processing and visualization code available
AIS Trajectories from Danish Waters for Abnormal Behavior Detection^25^	>100,000 unlabeled training trajectories; 521 labeled evaluation trajectories; 25 abnormal trajectories	Danish waters, including Sjælland and Bornholm; 2021; labeled evaluation on 13 Dec. 2021	whole-trajectory level; binary normal/abnormal labels; abnormal trajectories linked to a collision/search-and-rescue event and related traffic deviations	manual trajectory-level annotation; public DTU data collection; training and evaluation files available
Labeled AIS trajectories near Svalbard for abnormal behavior detection^26^	35,055 trajectories; 372 vessels; 16 anomalous trajectories (11 MELKART-5; 5 YAGRY)	Svalbard Arctic submarine-cable area; 2020–2022; anomaly labels centered on the Jan. 2022 cable-breach event	whole-trajectory level; binary anomaly labels; 2-min resampling; trajectory duration of 10 min–12 h; repeated cable-area crossings as the abnormal class	manual trajectory-level annotation; public DTU dataset; JSON and pickle formats available
Global Fishing Watch Apparent Fishing Effort^27^^,^^28^	22 billion AIS messages; >70,000 industrial fishing vessels	Global; 2012–2016 in the cited study corpus	AIS-position level; binary apparent-fishing/non-fishing predictions; aggregable to apparent fishing effort	CNN-based inference trained on expert-labeled examples; public data products and APIs; labels model-generated rather than manual ground truth
NCL-DASB^29^	Not reported	Tynemouth port, Newcastle, UK; period not reported	trajectory level; abnormal-behavior labels; class taxonomy and annotation granularity incompletely documented	labeled dataset and open-source annotation API reported; stable public dataset repository and complete label documentation not clearly identified
MarineCadastre/NOAA AIS^30^	Varies by release and region; cited 2024 product contains AIS-derived vessel tracks across U.S. waters	U.S. coastal and ocean waters; Jan.–Dec. 2024 for the cited release	track-level AIS resource; self-reported navigational status, including under way, at anchor, and moored; not independently annotated behavior labels	AIS self-reported status as weak labels; publicly downloadable from NOAA/MarineCadastre; not a manual behavior-annotation benchmark
Semi-supervised fishing behavior dataset^31^	600 trajectories; 535 training; 15 validation; 50 test trajectories	U.S. AIS from MarineCadastre; specific region and period not reported	sequence/message level; binary sailing/fishing labels; labels propagated from geometric clusters; time-series classification	clustering-derived pseudo-labels; source AIS data and code public; labels not independent manual ground truth
Manually annotated fishing trajectory dataset^32^	128 fishing-vessel trajectories; point-wise labels	Real-world fishing trajectories; region and period not reported	point level; binary fishing/non-fishing labels; segment-level reconstruction of sailing to fishing grounds, fishing, and returning to port	manual point-level annotation; dataset link provided by the first author; repository DOI and explicit license not reported

Note: Annotation unit denotes the object receiving a label: AIS position, sequence/window, whole trajectory, or incident-linked track. “Manual” denotes human-assigned or human-correlated labels; “weak label” denotes an operational or self-reported field without independent behavioral verification; “pseudo-label” denotes an algorithmically generated training label; and “model-generated” denotes an output inferred by a trained model. “Not reported” indicates information unavailable in the cited source. Public availability refers to the behavior dataset or derived label product rather than the underlying raw AIS source alone.

As shown in Table 2, the core problem of current annotated ship behavior data is not merely the limited number of datasets, but the simultaneous imbalance among openness, semantic hierarchy, and label reliability. First, existing public datasets are dominated by AIS as a single data source. Although VMS and LRIT are important for operational regulation and long-range offshore monitoring, reusable open behavior annotation benchmarks have not yet been established because of regulatory restrictions, commercial sensitivity, and legal constraints.^22^^,^^23^ Second, existing labeling systems generally remain task-specific and low in semantic granularity, mainly focusing on binary or small-category labels such as “normal/abnormal” and “fishing/non-fishing”. In contrast, annotations for high-level behavioral intention, multi-ship interaction relationships, navigation-rule compliance, and multi-granularity semantic processes remain insufficient. Third, a considerable proportion of labels in existing datasets are not manual behavioral ground truth in the strict sense, but are generated from AIS self-reported status, rule-based judgment, clustering-based pseudo-labeling, or model inference. Such labels can support training for specific tasks, but they cannot be regarded as authentic, unified, and transferable standards for behavioral semantics.^27^^,^^28^^,^^29^^,^^30^^,^^31^^,^^32^ Table 2 further shows that nominal dataset size is not directly comparable across resources because the annotation unit ranges from individual AIS positions to complete trajectories and incident-linked tracks. Availability must also be interpreted carefully: an open raw AIS archive, an open annotation tool, and an openly downloadable behavior-labeled benchmark provide different levels of reproducibility. MarineCadastre is therefore retained as an accessible weak-label resource rather than treated as equivalent to manually annotated ground truth, whereas the Global Fishing Watch product is explicitly identified as model-generated inference.

In other words, the main bottleneck in current ship navigation behavior pattern mining is no longer only insufficient algorithmic capability, but also the lack of a high-quality annotated data foundation with unified semantic definitions across scenarios, granularities, and tasks.

## Ship behavior modeling

Ship navigation behavior denotes a continuous spatiotemporal process formed by a ship in a specific navigational environment under the joint influence of traffic rules, environmental constraints, and maneuvering intentions.^3^^,^^33^^,^^34^ Traffic rules define the acceptable boundaries of ship motion, environmental constraints shape navigable space and feasible movement modes, and maneuvering intentions reflect task-driven and situation-aware decision-making. Therefore, ship behavior is not a simple mapping from trajectory geometry to behavioral labels. Rather, it is a composite spatiotemporal object jointly constituted by physical motion states, spatiotemporal interaction relationships among objects, and high-level semantic meanings of navigational events.^3^^,^^33^^,^^34^

According to how behavioral information is organized and how semantic abstraction is achieved in existing models, ship behavior modeling can be broadly summarized into three categories: segment mapping, hierarchical composition, and knowledge association.^35^

Segment-mapping-based modeling takes local trajectory segments as the basic analytical objects. Typical methods include multi-scale behavior analysis based on maritime areas or functional zones, time-window-based trajectory segmentation and classification, and Stop/Move trajectory segmentation.^36^^,^^37^ These methods essentially map trajectory segments directly to specific behavior states, categories, or local patterns, usually using geometric features such as speed, course, and position as the main inputs. They can characterize local motion changes but have difficulty representing complex behaviors with task semantics and interaction semantics, such as reconnaissance, exercises, and cooperative fishing.

Hierarchical-composition-based modeling attempts to organize behavior into layered structures, such as “atomic–topological–traffic” levels,^38^ thereby enabling a preliminary abstraction from motion states and spatial relationships to navigational events. This type of modeling partially overcomes the limitations of segment mapping. However, the connections between layers often rely on manually designed rules, topological relations, and compositional descriptions. Such models still cannot provide a unified representation that connects low-level continuous observations, multiple interaction relationships, and high-level semantic interpretation. As a result, they usually support only a limited set of navigational event modeling tasks.

Knowledge-association-based modeling is represented by ship behavior ontologies and activity knowledge graphs.^3^^,^^33^^,^^34^^,^^39^^,^^40^ These methods use predefined navigational events as the conceptual core and organize related information, such as time, location, and objects, into Semantic Web structures, graph structures, or semi-structured text. By emphasizing behavioral concept definitions and rule-constraint representation, they provide clear conceptual boundaries, explicit rule bases, and explainable reasoning processes. However, they are usually highly dependent on expert knowledge modeling, and their ability to characterize continuous motion-state evolution, interaction relationship dynamics, and cross-scenario transfer remains limited.

In summary, the fundamental gap in existing ship behavior modeling is not simply an insufficient ability to recognize local behaviors. Rather, it is the lack of a modeling framework that can uniformly represent continuous motion observations, spatiotemporal interaction processes, and high-level event semantics as components of the same behavioral object. Existing models can often output a behavior label, but they usually cannot further answer which local motion states and evolutionary processes constitute the behavior, which environmental objects or surrounding ships are involved in the spatiotemporal interaction, and according to which rules or knowledge the process is interpreted as a specific navigational event. For complex behavior recognition, novel behavior discovery, abnormal behavior detection, and rule-compliance analysis, classification results alone are insufficient. Models must also provide explanatory information that can support real-world decision-making. Moreover, ship behavior is not a closed and rigid standard pattern, but a dynamic process with spatiotemporal degrees of freedom formed by ship operators under rule constraints and environmental changes. Therefore, ship behavior modeling should be computable and explainable, as well as extensible and transferable to behavioral variations, new scenario objects, and new task semantics.

Based on this understanding, this review constructs a three-layer ship behavior model from the perspective of progressive abstraction, moving from physical motion states to spatiotemporal interaction relationships and then to navigational event semantics. Ship behavior is uniformly represented at three levels: physical behavior, interactive behavior, and semantic behavior. Unlike existing methods based on segment mapping, hierarchical composition, or knowledge association, this framework represents ship behavior as a computable, explainable, and extensible spatiotemporal semantic object. Within the same framework, continuous motion states, spatial relationship evolution, and high-level semantic inference are connected, thereby providing a unified modeling basis for complex behavior recognition, abnormal behavior explanation, and behavior similarity measurement in ship navigation behavior pattern mining, as shown in Figure 2.

**Figure 2:**
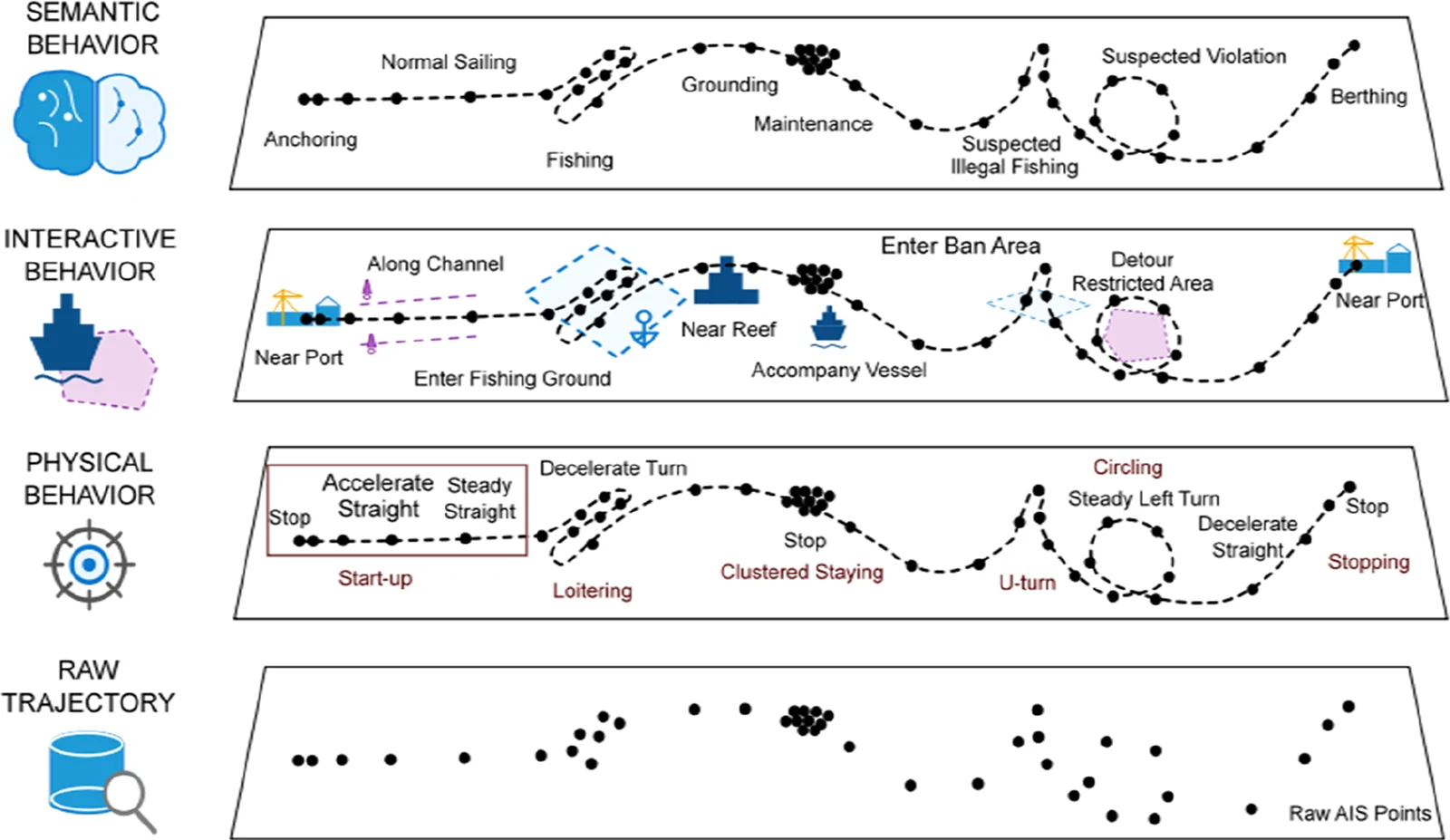
Three-layer spatiotemporal semantic modeling framework for ship behavior

Physical behavior refers to the physical motion process of a ship and its temporal evolution. This layer takes only the dynamic attribute sequence as input and characterizes local changes and persistent patterns of motion-related state variables, such as ship position, speed, course, and ROT. It does not introduce external contexts such as environmental objects, other ships, or navigation tasks.

Following the trajectory notation defined above, the dynamic-attribute sequence of vessel *v* over the trajectory-segment index interval [*i*,*j*] is defined as

$$D_{i:j}^{\left(v\right)}:={\left\langle D_{k}^{\left(v\right)}\right\rangle }_{k=i}^{j}\text{.}$$

The physical behavior associated with this segment can be schematically represented as

$$B_{i:j}^{\text{phy},\left(v\right)}=\Phi_{\text{phy}}\left(D_{i:j}^{\left(v\right)}\right)\text{,}$$

where *Φ*_phy_ denotes an abstract mapping from the dynamic-attribute sequence to a physical-behavior description. Depending on the analytical method, its output may be a deterministic state sequence, a multilabel description, or a probability distribution over candidate physical behaviors.

Dynamic attributes between adjacent sampling points, such as speed, course, and position, can characterize basic motion states, including acceleration, deceleration, constant-speed motion, left turn, right turn, straight navigation, and stop. The continuous temporal combination of these basic states, together with cumulative changes in trajectory morphology, further forms higher-order physical patterns, such as start-up, continuous turning, U-turn, loitering, circling, prolonged staying, and stable navigation. Physical behavior is therefore a multi-attribute motion description rather than a single mutually exclusive label. The same trajectory segment may simultaneously exhibit different motion states, such as acceleration and right turn, and their temporal combinations may form more complex physical patterns.

Interactive behavior describes the temporal evolution of spatial relations between a target vessel and the navigational environment or surrounding vessels. It identifies the objects involved in an interaction and the geographic context in which the behavior occurs. From the perspective of geographic information science and spatial analysis, interactive behavior can be represented through direction, distance, topology, and their joint temporal evolution.^41^^,^^42^

The geographic environmental objects in the study area, such as ports, channels, anchorages, protected areas, and control areas, are represented as the indexed collection

$$\begin{array}{cc} E={\left(e_{m}\right)}_{m=1}^{M}\text{,} & M=\text{card}\left(E\right)\text{.} \end{array}$$

At target-vessel time $t_{k}^{\left(v\right)}$, let $E_{k}^{\left(v\right)}\subseteq E$ denote the environmental objects considered relevant to vessel *v*, and let $N_{k}^{\left(v\right)}\subseteq V$ denote the surrounding vessels considered relevant at that time, with $v\notin N_{k}^{\left(v\right)}$. The set of candidate interaction objects is

$$O_{k}^{\left(v\right)}=E_{k}^{\left(v\right)}\cup N_{k}^{\left(v\right)}\text{.}$$

Environmental objects and surrounding vessels are treated as typed objects with persistent identifiers. For a surrounding vessel $u\in N_{k}^{\left(v\right)}$, its position or geometry must be temporally aligned to $t_{k}^{\left(v\right)}$. When no observation exists at exactly the same time, the interpolation, nearest-time matching, or state-estimation rule must be specified by the corresponding application.

For any object $o\in O_{k}^{\left(v\right)}$, let *g*_*k*_(*o*) denote its spatial geometry at $t_{k}^{\left(v\right)}$. The spatial relation between vessel *v* and object *o* is represented by the heterogeneous relation tuple

$$R_{k,o}^{\left(v\right)}=\left(\text{dir}\left(l_{k}^{\left(v\right)},g_{k}\left(o\right)\right),\text{dist}\left(l_{k}^{\left(v\right)},g_{k}\left(o\right)\right),\text{topo}\left(l_{k}^{\left(v\right)},g_{k}\left(o\right)\right)\right)\text{.}$$

The direction component takes an angular value from zero inclusive to 2*π* exclusive, or the undefined value ⊥; the distance component is nonnegative; and the topology component belongs to the predicate set $T_{o}$ admissible for the geometry type of object *o*. The relation tuple is not treated as an ordinary Euclidean feature vector because it combines continuous and categorical components.

Consistent with the trajectory notation, $l_{k}^{\left(v\right)}$ remains a geographic coordinate. For metric and planar directional calculations, let *Π*_*Ω*_ denote a coordinate transformation from geographic coordinates to a projected coordinate reference system selected for the spatial extent *Ω* of the study area:

$$\begin{array}{cc} {\tilde{l}}_{k}^{\left(v\right)}=\Pi_{\Omega }\left(l_{k}^{\left(v\right)}\right)=\left(x_{k}^{\left(v\right)},y_{k}^{\left(v\right)}\right)\in R^{2}\text{,} & {\tilde{g}}_{k}\left(o\right)=\Pi_{\Omega }\left(g_{k}\left(o\right)\right)\subseteq R^{2}\text{.} \end{array}$$

The projected coordinate system must be appropriate for the spatial extent of the analysis. For large-area or cross-zone trajectories, a single local projection should not be assumed without evaluating projection distortion.

Under the present formulation, the target vessel is represented by its AIS reference position, whereas the target-object geometry may be a point, polyline, or polygon. Extended vessel footprints or safety domains can be incorporated through geometry-to-geometry operators, but they are not assumed in the point-based definitions later in discussion.

For a nonempty projected target geometry ${\tilde{g}}_{k}\left(o\right)$, the distance relation is defined as

$$\text{dist}\left(l_{k}^{\left(v\right)},g_{k}\left(o\right)\right):=\min_{\left(x_{q},y_{q}\right)\in {\tilde{g}}_{k}\left(o\right)}\sqrt{{\left(x_{k}^{\left(v\right)}-x_{q}\right)}^{2}+{\left(y_{k}^{\left(v\right)}-y_{q}\right)}^{2}}\text{.}$$

Its unit is the linear unit of the projected coordinate system, normally meters. The value is zero when the vessel position lies on or within the target geometry. This is a point-to-geometry distance rather than a distance to the object boundary; if the latter is required, it must be calculated against $\partial {\tilde{g}}_{k}\left(o\right)$. For ship-ship interactions under the present point-based formulation, the operator reduces to the Euclidean distance between the two temporally aligned reference positions.

To define direction, let ${\tilde{r}}_{k,o}=\left(x_{k,o}^{\text{ref}},y_{k,o}^{\text{ref}}\right)$ denote a unique object reference point. For a point object, this is the projected object position; for a disjoint polyline or polygon, it may be the unique nearest point when that point is well defined. Provided that ${\tilde{r}}_{k,o}\neq {\tilde{l}}_{k}^{\left(v\right)}$, the absolute bearing from the target vessel to the object is

$$\text{dir}\left(l_{k}^{\left(v\right)},g_{k}\left(o\right)\right):=\beta_{k,o}^{\left(v\right)}=\mathrm{mod}\left(\mathrm{atan}\;2\left(x_{k,o}^{\text{ref}}-x_{k}^{\left(v\right)},y_{k,o}^{\text{ref}}-y_{k}^{\left(v\right)}\right),2\pi \right)\text{.}$$

The bearing is measured clockwise from the positive *y* axis, that is, grid north. If the vessel position and the reference point coincide, or if an extended object does not provide a unique and operationally meaningful reference point, the direction is set to ⊥. When the vessel lies on or within an extended object, an application-specific semantic reference, such as a designated entrance, exit, or operational control point, must be stated explicitly rather than selected arbitrarily. If the bearing is compared with AIS COG or HDG, grid-to-true-north convergence must either be corrected or shown to be negligible within the study area. When an object-relative direction is required, it may be defined as

$$\rho_{k,o}^{\left(v\right)}=\mathrm{mod}\left(\beta_{k,o}^{\left(v\right)}-\psi_{k}^{\left(v\right)}+\pi ,2\pi \right)-\pi \text{,}$$

where $\psi_{k}^{\left(v\right)}$ must be explicitly identified as either COG or HDG according to the application.

The topological relation is determined according to the Dimensionally Extended Nine-Intersection Model and the spatial predicates specified by the Open Geospatial Consortium.^41^^,^^42^ For a point-region interaction between the vessel position and a valid areal object, it is defined as

$$\text{topo}\left(l_{k}^{\left(v\right)},g_{k}\left(o\right)\right)=\left\{ \begin{array}{ll} \text{disjoint}, & {\tilde{l}}_{k}^{\left(v\right)}\notin {\tilde{g}}_{k}\left(o\right),\\ \text{touches}, & {\tilde{l}}_{k}^{\left(v\right)}\in \partial {\tilde{g}}_{k}\left(o\right),\\ \text{within}, & {\tilde{l}}_{k}^{\left(v\right)}\in \mathrm{int}\left({\tilde{g}}_{k}\left(o\right)\right). \end{array}\right.$$

For this point-region case, the value domain consists of the three predicates disjoint, touches, and within. Additional predicates and admissible argument orders depend on the dimensions and geometry types of the interacting objects. Geometry-to-geometry relations such as crosses or overlaps may require extended representations, whereas predicates such as equals or contains have geometry-type-specific applicability.

Because several objects may coexist at the same sampling time, object identity must be retained together with the relation value. The relation representation at $t_{k}^{\left(v\right)}$ is therefore defined as

$$R_{k}^{\left(v\right)}:o\mapsto R_{k,o}^{\left(v\right)},o\in O_{k}^{\left(v\right)}\text{.}$$

The corresponding time-stamped relation-evolution sequence over the trajectory segment is

$$R_{i:j}^{\left(v\right)}:={\left\langle \left(t_{k}^{\left(v\right)},R_{k}^{\left(v\right)}\right)\right\rangle }_{k=i}^{j}\text{.}$$

Retaining the actual timestamps is necessary because AIS observations are irregularly sampled and equal index differences do not imply equal elapsed times. The interactive behavior can then be represented as

$$B_{i:j}^{\mathrm{int},\left(v\right)}=\Phi_{\mathrm{int}}\left(B_{i:j}^{\text{phy},\left(v\right)},R_{i:j}^{\left(v\right)}\right)\text{,}$$

where *Φ*_*int*_ denotes an abstract mapping that generates a relational-state sequence, a multilabel interaction description, or a probability distribution over candidate interaction patterns.

Typical interactive behaviors include approaching, moving away, entering, leaving, navigating along, crossing, detouring, accompanying, encountering, and overtaking. For an underlying continuous trajectory, port entry involves a boundary crossing from the exterior to the interior of the port region. In discretely sampled AIS data, however, the observed predicate sequence may change directly from disjoint to within without containing an explicit touches observation. Boundary crossing should therefore be inferred from consecutive trajectory segments, interpolation, or a stated spatial tolerance rather than from point predicates alone. Before entry, the process may also be characterized by decreasing distance and a bearing directed toward a designated port entrance or interior reference. The joint temporal evolution of direction, distance, and topology describes not only whether a vessel intersects an object or region, but also how it approaches, enters, traverses, and leaves it.

Semantic behavior refers to a context-conditioned interpretation formed from physical behavior, interactive behavior, vessel attributes, voyage-related context, and maritime domain knowledge. It represents event, intention, anomaly, or compliance hypotheses rather than low-level motion alone. Define the voyage-related sequence associated with the segment as

$$V_{i:j}^{\left(v\right)}:={\left\langle V_{k}^{\left(v\right)}\right\rangle }_{k=i}^{j}\text{.}$$

The semantic behavior is represented as

$$B_{i:j}^{\text{sem},\left(v\right)}=\Phi_{\text{sem}}\left(B_{i:j}^{\text{phy},\left(v\right)},B_{i:j}^{\mathrm{int},\left(v\right)},R_{i:j}^{\left(v\right)},S^{\left(v\right)},V_{i:j}^{\left(v\right)},K\right)\text{,}$$

where *Φ*_sem_ denotes an abstract semantic-inference mapping and K denotes domain knowledge, including maritime rules, navigational experience, regional control requirements, historical behavior patterns, and scenario constraints. Depending on the inference method and available evidence, $B_{i:j}^{\text{sem},\left(v\right)}$ may be a single hypothesis, a set of candidate hypotheses, or a probability distribution over hypotheses. Typical outputs include port entry or departure, berthing, anchoring, overtaking, suspected illegal entry, abnormal staying, and suspicious wandering. Risk estimates and regulatory decisions are treated as downstream outputs generated by separate risk or decision models rather than as primitive semantic-behavior categories.

By preserving the vessel identifier *v*, the index interval [*i*,*j*], the observation times, and the object-relation pairs in $R_{i:j}^{\left(v\right)}$, a semantic hypothesis can be linked to the corresponding trajectory segment and traced to its constituent physical states, interaction objects, spatial-relation evolution, and supporting domain knowledge. This structure provides the basis for an evidence-linked explanation chain without assuming that the conceptual framework itself constitutes an empirically validated end-to-end architecture.

Conceptually, the proposed three-layer framework represents ship behavior as a computable, explainable, and extensible spatiotemporal semantic object, rather than merely as a label-discrimination target. The physical behavior layer corresponds to kinematic primitives derived from a target vessel’s motion states. The interactive behavior layer corresponds to relational or contextual patterns involving another vessel, a region, a route, a facility, or another explicit reference object. The semantic behavior layer combines those observations with vessel attributes, voyage context, operational procedures, maritime rules, and domain knowledge to form event, intention, anomaly, or compliance hypotheses. Aggregation scale is an independent dimension: single-vessel, pairwise or multi-agent, and regional or collective analyses may occur at different representation levels. Regional traffic density, channel extraction, and bottleneck identification are aggregate traffic structures rather than higher-level behavior semantics, while collision risk, accident risk, and regulatory decisions are downstream uses of behavior evidence rather than behavior layers. Individual methods may contribute to more than one layer, so the framework is intended as an integrative conceptual structure for organizing existing research, not as a formal ontology or an empirically validated end-to-end computational architecture.

## Ship behavior pattern mining methods

Ship navigation behavior pattern mining aims to reveal the spatiotemporal regularities and latent event semantics of ship behavior from trajectory observations and associated environmental data using spatiotemporal analysis, data mining, and artificial intelligence.^19^^,^^35^ It includes behavior recognition and regularity discovery from historical trajectories, as well as real-time evolution analysis and future trend inference during navigation.^19^^,^^37^^,^^38^ To support a consistent synthesis, this Review organizes the literature into four analytical groups: prior-knowledge-driven methods, feature-based statistical learning methods, deep representation learning methods, and large-model-enhanced methods. This organization is intentionally non-exclusive. The first three groups describe primary representation-and-inference paradigms, whereas large-model enhancement is a cross-cutting configuration that may retrieve knowledge, coordinate tools, or synthesize outputs generated by the other paradigms. The groups should therefore be compared along common design dimensions rather than interpreted as a linear sequence of technical replacement. The data sources, methodological system, and applications of ship navigation behavior pattern mining are summarized in Figure 3.

**Figure 3:**
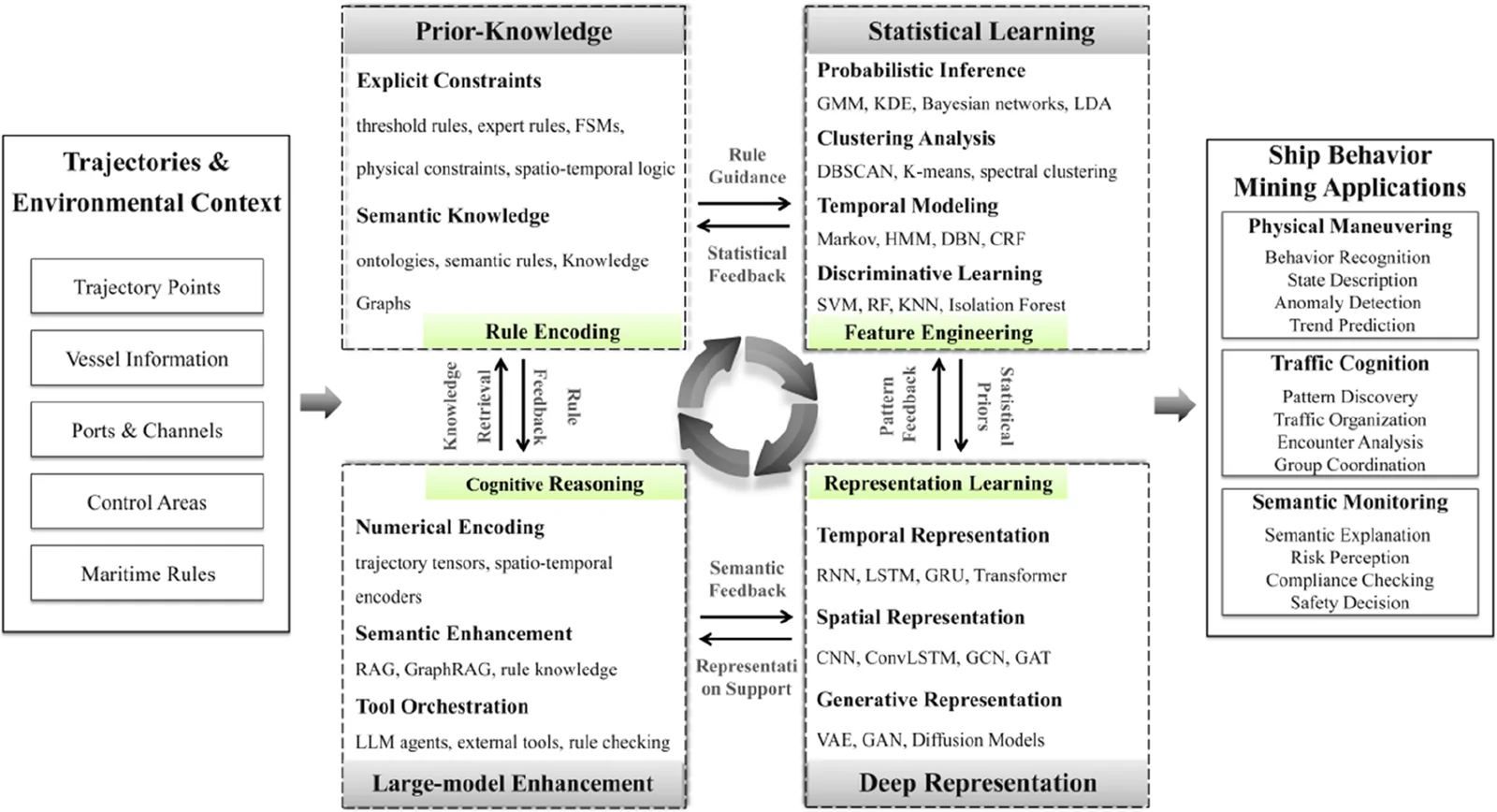
Data, methodological system, and applications of ship behavior mining

### Prior-knowledge-driven methods

Prior-knowledge-driven methods usually formalize maritime regulations, expert experience, or domain knowledge as computable logical rules, formula-based equations, or semantic symbols, and then identify ship behavior patterns or typical navigational events through explicit-constraint-based discrimination or semantic knowledge matching.^3^^,^^33^^,^^34^^,^^37^^,^^38^ Explicit-constraint-based discrimination methods generally use local motion states or spatiotemporal positional relationships to detect behavior patterns from low-level observable features in a bottom-up manner. By contrast, semantic knowledge matching methods construct knowledge structures, such as event concept ontologies or spatiotemporal semantic relations of behavior, and identify behavior events or evolutionary processes of interest in a top-down manner.

#### Explicit-constraint-based methods

Explicit-constraint-based discrimination methods detect ship behavior patterns by constructing explicit logical rules or constraint conditions for local motion states and spatiotemporal interactive relationships. In essence, these methods transform fragmented prior knowledge, such as maritime experience, into “IF–THEN” behavior reasoning rules. They usually first measure physical motion features and spatiotemporal relationships, and then construct behavior discrimination conditions through rule matching or conditional judgment. Typical studies include behavior judgment methods based on expert rules and threshold rules,^43^^,^^44^^,^^45^^,^^46^ as well as methods that describe behavioral state evolution through state organization and event composition mechanisms.^37^^,^^38^ To improve model adaptability, some studies further introduce fuzzy logic and probabilistic methods to represent uncertain boundaries, and combine kinematic constraints to improve rule consistency.^47^^,^^48^^,^^49^^,^^50^^,^^51^

#### Semantic-knowledge-based methods

Semantic knowledge matching methods use predefined concepts, relations, properties, and constraints to represent ship behavior patterns and associated objects in structured and queryable forms.

Existing studies mainly include ontology reasoning methods,^3^^,^^33^^,^^34^ semantic rule methods,^3^^,^^33^^,^^34^ and knowledge graph methods.^39^^,^^40^ Ontology reasoning methods use description logic to define entity hierarchies and constraint axioms, thereby supporting the formal description of behavior ontologies and semantic consistency checking. Semantic rule methods use representation frameworks and rule languages such as the Resource Description Framework (RDF), Web Ontology Language (OWL), and Semantic Web Rule Language (SWRL) to construct entity relations and reasoning rules. Through Semantic Web structures, these methods support behavior reasoning and constraint verification, thereby enabling behavior recognition and anomaly detection. Knowledge graph methods model ships, trajectories, behavior events, maritime rules, and other prior objects of interest as conceptual relation networks, and combine graph queries with path reasoning to mine complex behavior patterns. During knowledge construction, trajectory observation data usually need to be abstracted into predefined knowledge objects, such as Stop-Move patterns, event quintuples consisting of subject, behavior, object, time, and space, and semantically annotated trajectories. These abstractions enable semantic representation of trajectory information and provide basic inputs for knowledge graph construction and behavior pattern ontology reasoning.^37^^,^^39^^,^^40^ Recent port-oriented research has demonstrated the effectiveness of integrating a maritime knowledge graph with graph neural networks for ship-speed forecasting, showing how structured relations among ships, port infrastructure, and operational context can enhance data-driven graph representation and context-aware prediction.^7^

Overall, prior-knowledge-driven methods have the advantages of clear structure, strong interpretability, and high consistency with maritime rules. However, they heavily depend on the completeness and accuracy of manually constructed entities, relations, and properties. They are therefore often difficult to adapt to complex and open maritime environments, and may lead to rule conflicts, state explosion, and high knowledge maintenance costs. Accordingly, these methods are more suitable as explicit constraint and semantic reasoning modules that provide prior knowledge and structured constraints for data-driven methods, thereby improving the interpretability and reliability of ship navigation behavior pattern mining.

### Feature-based statistical learning methods

Feature-based statistical learning methods learn the latent structures and evolutionary regularities of ship behavior in a data-driven manner through feature extraction and statistical modeling of ship trajectories.^52^^,^^53^ According to differences in how the spatial distributions and temporal relationships of latent trajectory features are modeled, existing studies can be broadly grouped into four categories: probabilistic inference methods, clustering analysis methods, temporal modeling methods, and discriminative learning methods.^52^^,^^53^

#### Probabilistic inference models

Probabilistic inference methods model uncertainty and latent structures in ship behavior by establishing probabilistic distribution relationships between trajectory data and behavior states.^52^^,^^53^^,^^54^^,^^55^ These methods usually use probabilistic models to describe statistical associations between ship trajectory observations and behavior states, and then perform behavior state recognition or risk assessment through parameter estimation and probabilistic inference. Typical methods include Gaussian mixture model (GMM), kernel density estimation (KDE),^54^ Bayesian networks,^55^ and latent Dirichlet allocation (LDA).^56^ Among them, GMM characterizes the distribution of complex behavior patterns through a combination of multiple Gaussian distributions, KDE is commonly used for spatial density estimation and hotspot analysis, and Bayesian networks model associations among behavior variables through probabilistic dependencies.^54^^,^^55^

#### Clustering analysis models

Clustering analysis methods measure spatial, temporal, and feature similarities among trajectory samples to mine latent ship behavior patterns under unsupervised conditions.^36^^,^^57^^,^^58^^,^^59^^,^^60^ According to how similarity relationships are characterized, related studies can be further divided into density-based clustering, distance-based clustering, and multivariate association clustering.^36^^,^^57^^,^^58^^,^^59^^,^^60^ Density-based clustering methods extract trajectory aggregation areas using local density distributions, with typical methods including density-based spatial clustering of applications with noise (DBSCAN)^57^^,^^58^ and hierarchical density-based spatial clustering of applications with noise (HDBSCAN).^59^ Distance-based clustering methods divide behavior patterns according to spatial distance measures or customized distances in feature space, with typical methods including k-means, spectral clustering, and trajectory clustering.^36^^,^^60^ Multivariate association clustering methods discover latent behavioral regularities by mining frequent behavior sequences and co-occurrence relationships, with typical methods including Apriori-based association rule mining.^61^

#### Temporal modeling methods

Temporal modeling methods focus on state-transition dependencies among trajectory points or trajectory segments, and model the dynamic evolution of ship behavior patterns.^3^^,^^22^^,^^62^ These methods usually use probabilistic transition relationships among trajectory sequence states to describe the temporal evolution of behavioral features, thereby supporting continuous behavior pattern recognition and future state prediction. Typical methods include Markov chains and their extensions, such as hidden Markov model (HMM),^22^ dynamic Bayesian network (DBN),^3^ and conditional random field (CRF).^62^ Markov models describe behavior evolution processes through finite state-transition relationships; HMM infers behavior patterns by introducing hidden states; DBN uses dynamic probabilistic graph structures to model complex temporal dependencies among trajectory features; and CRF supports behavior sequence labeling through conditional probability modeling.^3^^,^^22^^,^^62^

#### Discriminative learning models

Discriminative learning methods learn mapping relationships between trajectory features and behavior labels to support ship behavior classification, state recognition, and anomaly detection.^63^^,^^64^^,^^65^^,^^66^ Unlike probabilistic inference methods, which emphasize the generation probability of behavior, discriminative learning methods directly construct decision boundaries in the feature space and optimize classification performance for behavior discrimination. Typical methods include support vector machine (SVM), random forest (RF), k-nearest neighbors (KNN), and logistic regression (LR).^63^^,^^64^^,^^65^ SVM constructs classification hyperplanes based on the maximum-margin principle, RF performs nonlinear classification of complex behavior patterns through ensembles of decision trees, and KNN identifies behavior patterns using local neighborhood information.^63^^,^^64^^,^^65^ In abnormal behavior detection, isolation forest and one-class SVM are also commonly adopted.^13^^,^^40^^,^^66^^,^^67^

Overall, feature-based statistical learning methods support structural discovery and discriminative analysis of ship behavior patterns through explicit feature representation and statistical modeling of ship trajectories.^52^^,^^63^ These methods essentially follow a “feature representation–statistical modeling–pattern discrimination” learning paradigm.^52^^,^^53^^,^^63^ By modeling kinematic features, spatial interaction relationships, and sequential statistical regularities, they attempt to explicitly characterize behavior patterns. Compared with prior-knowledge-driven methods, they reduce dependence on manual rules and semantic knowledge,^52^^,^^53^ and emphasize automatic learning of behavior distributions and latent patterns from feature information. However, their representational capability remains mainly constrained by feature extraction and the validity of statistical assumptions, which limits their ability to characterize complex semantic behavior. Therefore, within the overall behavior mining framework, these methods usually serve as a basic modeling paradigm, providing structured inputs and statistical priors for deep representation learning and cognitive reasoning methods.

### Deep representation learning methods

Deep representation learning methods automatically learn latent representations from ship trajectory sequences, multi-ship interaction relationships, and trajectory-environment dependencies through neural networks, thereby reducing dependence on manually designed features and prior constraints. Unlike feature-based statistical learning methods, which rely on explicit feature construction and statistical assumptions, these methods can learn deeper semantic features from trajectory data through multilayer nonlinear mappings. According to differences in how spatiotemporal features are learned, existing studies can be broadly grouped into temporal-dependency learning, spatial-structure learning, and generative learning methods.^17^^,^^18^^,^^68^ Temporal-dependency learning and spatial-structure learning belong to discriminative representation learning and are used to model behavioral evolution regularities and spatial association structures, respectively. Generative learning methods, by contrast, learn latent distributions of behavior data to construct implicit behavior representations.

#### Temporal-dependency learning methods

Temporal-dependency learning methods treat ship trajectories as time-ordered multivariate sequences and learn hidden patterns in ship motion-state changes through recurrent structures, convolutional structures, or attention mechanisms. Their core objective is not simply to predict future positions, but to extract temporal features from continuous trajectories that can represent behavior states and behavior transitions. Typical models include recurrent neural network (RNN), long short-term memory (LSTM), and gated recurrent unit (GRU) architectures,^68^^,^^69^^,^^70^ which are used to encode recursive state evolution in trajectories. Recent studies often use convolutional structures to extract short-term local dynamic patterns,^71^^,^^72^ and then combine LSTM, GRU, Transformer, and related models to capture long-range temporal dependencies and perform weighted fusion of local spatial features from key temporal segments.^69^^,^^70^^,^^73^^,^^74^

#### Spatial-structure learning methods

Spatial-structure learning methods treat ship trajectories as spatial line features and model spatial distribution relationships through grids or graph structures. These methods usually extract behavior features using local neighborhood information, spatial topological relations, or inter-object interaction relations, thereby enabling deep representation of ship behavior distributions. In grid-based modeling, typical methods include convolution-based trajectory image or raster modeling,^71^ convolutional long short-term memory (ConvLSTM),^72^ and local attention-enhanced models.^74^ These methods aggregate features through spatial grids or local temporal windows to capture local motion patterns. In graph-structure modeling, ships and environmental objects are usually represented as graph nodes, whereas spatial proximity relations, relative motion relations, and route connectivity relations are represented as graph edges. Graph neural networks are then used to learn interactive representations in non-Euclidean spaces. Typical models include graph attention network-long short-term memory (GAT-LSTM)-based attention models,^75^ spatiotemporal graph convolutional models such as ST-GRUA,^76^ and spatiotemporal graph neural networks for abnormal behavior detection, such as STGVAD.^77^

#### Generative representation methods

Generative representation methods learn the latent distribution structure of ship trajectory data and use latent-variable spaces to represent the behavior patterns embedded in trajectory data. Unlike discriminative methods, which directly learn mapping relationships for behavior classification or prediction, generative methods place greater emphasis on the trajectory data generation mechanism and the construction of latent semantic spaces. Typical methods include variational autoencoder (VAE) models and their sequence extensions,^70^^,^^78^ as well as generative adversarial network (GAN)-based models.^79^^,^^80^ VAE-based methods construct dense latent-space representations of trajectory data through probabilistic encoding-decoding mechanisms, whereas GAN-based methods learn generation mechanisms for multi-ship interaction trajectories and complex behavior patterns through adversarial learning.

Overall, deep representation learning methods weaken the dependence on rules and semantic constraints in prior-knowledge-driven methods, and further reduce the limitations of manually designed features and statistical assumptions compared with feature-based statistical learning methods. They therefore mark a shift from explicit shallow modeling to implicit deep learning.^17^^,^^18^^,^^68^^,^^69^^,^^70^^,^^71^^,^^72^^,^^73^^,^^74^^,^^75^^,^^76^^,^^77^^,^^78^^,^^79^^,^^80^ In ship behavior modeling, these methods serve as an intermediate representation layer connecting candidate relation modeling, feature-based statistical learning, and high-level cognitive reasoning. However, their interpretability and explicit semantic representation capability remain limited, and they usually need prior knowledge to introduce inductive biases.

### Large-model-enhanced methods

Large-model-enhanced methods are treated here as a cross-cutting system configuration rather than as a direct replacement for prior-knowledge, statistical, or deep representation methods. With the improvement of large language models (LLMs) in natural language understanding, knowledge organization, complex reasoning, and tool use, their potential role in ship navigation behavior pattern mining has gradually attracted attention.^81^^,^^82^^,^^83^^,^^84^^,^^85^^,^^86^ Unlike traditional trajectory prediction models or deep representation models, LLMs are not suitable for directly replacing low-level operations such as trajectory cleaning, motion-feature calculation, spatial-relation judgment, or numerical collision-risk computation.^82^^,^^83^^,^^84^^,^^85^^,^^86^ A more reasonable role is to use them as cognitive enhancement modules that establish semantic connections among trajectory analysis results, maritime rules, environmental objects, historical cases, and regulatory requirements. In this way, LLMs can assist complex behavior interpretation, rule-consistency checking, risk assessment, and decision-report generation.^83^^,^^84^^,^^85^^,^^86^^,^^87^^,^^88^ However, because LLMs mainly generate outputs based on language patterns, their outputs do not naturally satisfy the consistency requirements of trajectory evidence, physical constraints, and maritime rules. They may even generate behavior explanations that appear plausible but lack factual support.^82^^,^^84^^,^^85^^,^^86^^,^^87^^,^^88^ Therefore, large-model-enhanced ship navigation behavior pattern mining should still be regarded as a frontier exploratory direction. Its key issue is not simply to make LLMs “understand trajectories”, but to achieve verifiable, traceable, and constrained behavioral semantic reasoning under the joint support of computable trajectory features, structured knowledge, rule constraints, and external tools.^83^^,^^84^^,^^85^^,^^86^^,^^87^^,^^88^^,^^89^^,^^90^ According to the degree of integration between Transformer architectures and continuous trajectory values, as well as their collaboration mechanisms, existing explorations can be discussed along three potential technical pathways: native numerical representation, semi-structured semantic enhancement, and heterogeneous scheduling and collaboration.

#### Native numerical spatiotemporal trajectory large models

Native numerical spatiotemporal trajectory large models attempt to directly align continuous spatiotemporal features with the Transformer layers of large models.^82^^,^^86^^,^^89^^,^^90^ Because mainstream open-source LLMs, such as the Llama, Qwen, and DeepSeek series, are designed for discrete natural language, their tokenization mechanisms may disrupt the topological relationships and distance constraints of physical trajectories in continuous metric space. Therefore, such methods usually replace the standard textual token embedding layer of the large model with deep learning models such as multilayer perceptrons (MLPs) or spatiotemporal graph encoders, which project high-frequency trajectory tensors directly into a latent feature space. Furthermore, by freezing the backbone parameters and introducing parameter-efficient fine-tuning techniques such as low-rank adaptation (LoRA), these methods can reduce training costs while adapting large models to specific tasks such as trajectory representation, behavior recognition, or trajectory prediction.^81^^,^^86^^,^^89^ However, a severe bottleneck remains: there is still a lack of spatiotemporal large models that are native to continuous trajectory data and possess low-level spatial geometry and calculus-based computational capability. Most existing end-to-end solutions are forms of cross-domain fine-tuning based on text-oriented LLMs, and thus have difficulty self-consistently learning and understanding features such as high-frequency nonlinear maneuvering embedded in ship trajectories.

#### Semi-structured knowledge-enhanced reasoning methods

In response to the high computational cost of native spatiotemporal large models and the lack of deep numerical computing capability, semi-structured knowledge-enhanced reasoning methods use other models to calculate physical motion features, identify interactive relationships, and extract navigation rules, thereby transforming continuous trajectories into semi-structured data that provide LLMs with cognitive inputs rich in hierarchical semantics.^83^^,^^84^^,^^85^^,^^86^^,^^87^^,^^88^ This paradigm helps bridge the gap between low-level numerical data and high-level semantics. On the one hand, hidden navigation patterns and relevant rules, such as the Convention on the International Regulations for Preventing Collisions at Sea (COLREGs), can be dynamically attached to the reasoning context as external priors.^34^^,^^36^^,^^39^^,^^40^^,^^87^ On the other hand, complex ship trajectories can be transformed into knowledge structures with both storage-management and reasoning potential, such as graph retrieval-augmented generation (GraphRAG) nodes and vector knowledge bases.^83^^,^^84^^,^^87^ However, this type of method must introduce strict semantic risk quantification mechanisms for behavior explanations generated by LLMs. Logical mismatch with maritime rules, omission of key spatiotemporal events, and contextual redundancy should be jointly considered to ensure that machine-generated supervisory conclusions have rigorous legal and physical support.^84^^,^^85^^,^^86^^,^^87^^,^^88^

#### Large-model-orchestrated tool-augmented neuro-symbolic collaboration

Because autoregressive mechanisms have fundamental limitations in precise spatial geometric computation, such as Closest Point of Approach (CPA) and Time to Closest Point of Approach (TCPA), large-model-orchestrated neuro-symbolic collaboration upgrades the LLM into a “cognitive hub” for task orchestration, while decoupling traditional numerical trajectory analysis algorithms as execution agents or tools.^43^^,^^44^^,^^45^^,^^84^^,^^85^ When facing complex regulatory instructions, the LLM autonomously plans the task workflow, schedules external computable components for precise numerical processing, and finally integrates multi-source heterogeneous evidence to generate assessment reports. This heterogeneous scheduling and orchestration paradigm, in which the large model conducts implicit logical reasoning and smaller models or tools perform precise numerical computation, combines the rigor of traditional algorithms with the generalized explanatory capability of LLMs. At the current stage, it is a relatively mature pathway for avoiding the numerical computation weaknesses of LLMs and supporting practical implementation.^84^^,^^85^^,^^87^^,^^88^

Overall, large-model-enhanced methods are best understood as cross-cutting cognitive and orchestration components rather than self-sufficient replacements for numerical trajectory analysis, statistical inference, or symbolic reasoning. Their comparative advantage lies in connecting continuous trajectory evidence, maritime knowledge, retrieval sources, and external tools at the semantic interface.^83^^,^^84^^,^^85^^,^^86^^,^^87^^,^^88^^,^^89^^,^^90^ This advantage is conditional: modality mismatch can yield physically inconsistent outputs; long multi-ship contexts can create substantial latency; and domain-specific instruction data and evaluation benchmarks remain scarce.^82^^,^^84^^,^^85^^,^^86^^,^^87^^,^^88^^,^^89^^,^^90^ Future systems should therefore implement a verified “generation–detection–repair” loop in which numerical and rule-based components produce or check evidence, the large model synthesizes candidate interpretations, and explicit validators identify logical mismatch, missing elements, and unsupported claims before decision support is delivered.

Table 3 compares the four analytical groups using eight shared dimensions: methodological role, knowledge and supervision requirements, uncertainty treatment, interpretability and traceability, transferability and dominant failure modes, computational and engineering profile, suitable behavior representations and tasks, and priority validation evidence. The comparison distinguishes what each group computes from how it should be evaluated and explicitly links method selection to the physical, interactive, and semantic representations defined above.

**Table 3:** Cross-paradigm comparison of four analytical groups in ship behavior mining

Comparison dimension	Prior-knowledge-driven methods	Feature-based statistical learning methods	Deep representation learning methods	Large-model-enhanced methods
Methodological status and primary role	primary representation-and-inference paradigm. Encodes expert rules, physical constraints, ontologies, or semantic relations and can operate as a standalone reasoner or a constraint module.	primary representation-and-inference paradigm. Uses engineered features and statistical assumptions to estimate distributions, transitions, clusters, or decision boundaries.	primary representation-and-inference paradigm. Learns latent sequence, grid, graph, or generative representations through neural optimization.	cross-cutting integration configuration rather than a direct replacement for the other three. Supports retrieval, semantic synthesis, and tool orchestration around numerical or symbolic components.
Knowledge, data, and supervision requirements	high domain-knowledge and maintenance demand; generally low-to-moderate labeled-data demand.	moderate domain expertise for feature design and assumptions; label demand ranges from none in clustering to task-specific labels in discriminative learning.	high representative-data demand; supervision may be labeled, self-supervised, weakly supervised, or synthetic. Domain priors are optional but often beneficial.	high and heterogeneous requirements: trajectory-derived inputs, curated knowledge and retrieval sources, instruction or alignment data, and reliable external tools.
Uncertainty treatment	explicit when fuzzy logic, probabilities, or rule confidence are modeled; deterministic thresholds otherwise hide uncertainty.	often supports likelihoods, posteriors, confidence scores, or state probabilities, but calibration depends on model assumptions and data representativeness.	can use probabilistic outputs, ensembles, Bayesian approximations, or generative distributions; overconfidence and poor calibration remain common.	may express uncertainty linguistically, but verbal confidence is not calibrated evidence; uncertainty should be supplied by upstream models and explicit verification.
Interpretability and evidence traceability	usually high because rules, axioms, and reasoning paths are inspectable; traceability degrades when rule provenance or conflict resolution is unspecified.	moderate: features and statistical structure are inspectable, but individual decisions and nonlinear interactions may remain model-dependent.	low to moderate: latent features are difficult to interpret, and post-hoc explanations are not necessarily faithful.	potentially strong at the interface, but generated rationales are not inherently faithful; traceability requires source provenance, tool logs, and evidence grounding.
Transferability and dominant failure modes	limited by rule completeness, regional regulations, and scenario-specific knowledge; fails on novel, incomplete, or conflicting situations.	sensitive to feature validity, sampling bias, statistical assumptions, and distribution shift; may miss complex nonlinear interactions.	stronger within covered distributions but vulnerable to domain shift, sparse labels, spurious correlations, and physical or rule violations.	task-flexible but vulnerable to modality mismatch, hallucination, prompt and tool sensitivity, and scarce maritime benchmarks.
Computational and engineering profile	low online computation; high offline knowledge engineering, rule testing, and maintenance.	generally moderate training and inference cost; requires stable feature pipelines and periodic model updating.	high training and inference cost for long sequences, dense spatial encodings, and multi-agent graphs; hardware and latency constraints are significant.	high system-level cost from model inference, retrieval, context construction, and tool coordination; reproducibility, privacy, and latency require control.
Suitable behavior representations and tasks	rule-constrained events, compliance checking, interpretable anomaly hypotheses, and semantic verification; applicable across layers when knowledge is explicit.	state recognition, trajectory clustering, sequential labeling, and anomaly scoring; strongest for physical and interactive representations and bounded semantic tasks.	complex temporal and multi-agent representation, behavior recognition, interaction modeling, anomaly detection, and prediction-supported encoding; may support all layers with sufficient evidence.	evidence synthesis, semantic hypothesis generation, rule querying, report generation, and workflow orchestration; unsuitable as a standalone substitute for low-level geometry or physics.
Priority validation evidence	rule coverage and consistency, threshold sensitivity, expert agreement, false-alarm and missed-detection analysis, and cross-region applicability.	external test performance, calibration, feature ablation, robustness to missingness and sampling changes, and statistical-assumption checks.	cross-area and cross-vessel tests, uncertainty calibration, physical and rule-consistency checks, ablation, out-of-distribution tests, and latency.	evidence fidelity, retrieval and tool-call correctness, rule consistency, hallucination rate, reproducibility, human-review burden, and end-to-end latency.

The comparison yields four method-selection principles. First, tasks dominated by explicit regulatory constraints, auditability, or sparse labels should use prior-knowledge modules as the controlling layer. Second, when behavior can be represented by stable and interpretable features within a bounded operating domain, statistical learning offers the most favorable balance of efficiency, data demand, and transparency. Third, deep representation learning is justified when long-range temporal or multi-agent dependencies cannot be adequately captured by engineered features, but its use requires external-domain testing, calibration, and physical and rule-consistency checks. Fourth, large-model enhancement should be introduced only when the task requires knowledge retrieval, cross-modal semantic integration, or tool coordination; it should wrap verified numerical and symbolic components rather than replace them. Hybrid architectures should therefore assign different functions to the four groups instead of treating them as competing stages of a single technological hierarchy.

## Applications of ship behavior pattern mining

The ultimate goal of ship navigation behavior pattern mining is to transform ship trajectory analysis results into decision-relevant information for maritime applications. Based on the three-layer ship behavior modeling framework proposed above—physical behavior, interactive behavior, and semantic behavior—this section organizes applications according to the spatiotemporal information represented at each behavioral layer. Three major application categories are identified: physical maneuvering analysis, collective interaction cognition, and semantic cognitive monitoring. This application structure reflects a progressive transition from single-ship motion-state description to multi-ship traffic organization analysis, and further to event-level semantic understanding and decision inference.

### Physical maneuvering analysis

Physical maneuvering analysis is mainly oriented toward the analysis of single-ship motion states and basic maneuvering behaviors. Its core question is how a ship is moving. This category corresponds to the physical behavior layer in ship behavior modeling, which provides basic state-description information. Applications at this level focus on the representation of an individual ship’s motion states and their temporal changes, and are used to transform continuous ship observation trajectories into structured behavior sequences. Typical applications include navigation-state recognition, such as sailing and drifting; basic maneuvering behavior recognition, such as acceleration, deceleration, and U-turns; and ship trajectory segmentation and behavior monitoring.^36^^,^^37^^,^^46^^,^^71^ These applications provide standardized low-level behavior description units for maritime systems and serve as foundational information for subsequent complex behavior analysis and traffic cognition.

### Collective interaction cognition

Collective interaction cognition is mainly oriented toward multi-ship interaction relationships and regional traffic organization analysis. Its core question is how ships interact with each other and how the shipping network operates within a specified sea area. This category corresponds to the collective behavior description supported by the interactive behavior layer in ship behavior modeling. Related applications include channel extraction, traffic flow structure analysis,^91^^,^^92^ recognition of anchorage and channel usage patterns, ship aggregation behavior analysis,^93^ regional traffic density analysis,^94^ and bottleneck identification. In complex waters such as ports and straits, this category also involves port-entry and port-departure pattern analysis, multi-ship cooperative behavior analysis, and regional traffic evolution analysis. By mining traffic organization structures and operational regularities from massive multi-ship trajectory data, collective interaction cognition provides region- and group-oriented decision support for route planning, port scheduling, and regional navigation organization. At the regional interaction level, real-time accident prediction in narrow waterways and space-time-prism-based analysis have been used to characterize evolving multi-ship risks.^95^^,^^96^^,^^97^ Trajectory-derived operational patterns also provide an important foundation for sustainability-oriented applications, including high-resolution spatiotemporal ship-carbon-emission assessment and the systematic evaluation of low-carbon shipping pathways.^8^

### Semantic cognitive monitoring

Semantic cognitive monitoring integrates behavioral representations with environmental context and maritime knowledge to infer navigational intentions, interpret abnormal or rule-related events, and support risk-informed decision-making.^3^^,^^33^^,^^34^^,^^43^^,^^44^^,^^45^^,^^46^^,^^47^^,^^48^^,^^49^^,^^50^^,^^51^^,^^98^^,^^99^ Representative applications include semantic behavior recognition, intention inference, anomaly analysis, collision-risk diagnosis, and complex-event reasoning. Its principal function is to translate observed motion and interaction patterns into context-dependent event interpretations and operationally relevant evidence.

Recognized behavior patterns should not be treated as direct proxies for accident occurrence. Rather, they provide time-varying evidence for dynamic risk assessment. Kinematic changes and interaction patterns may be combined with channel geometry, traffic conditions, navigational rules, environmental factors, and established risk indicators to update estimates of collision, grounding, near-miss, or rule-violation risk. These estimates can inform proportionate interventions and subsequent feedback-based reassessment. Thus, behavior mining supports risk assessment and prevention by supplying structured, interpretable evidence, while final risk judgments and interventions remain dependent on dedicated risk models and broader situational information.

## Challenges and future directions

Ship navigation behavior pattern mining is moving from trajectory-driven pattern discovery toward trustworthy behavior understanding in complex maritime scenarios.^19^^,^^32^^,^^82^^,^^83^^,^^84^^,^^85^^,^^86^^,^^87^^,^^88^^,^^89^^,^^90^ With the development of large models and deep generative techniques, the bottleneck in this field is no longer limited to the recognition accuracy of individual models. Instead, challenges arise simultaneously at multiple levels: at the data level, behavior information remains difficult to organize, retrieve, and update; at the model level, physical constraints and multi-ship interaction mechanisms are still insufficiently incorporated; at the semantic level, contextual ambiguity and semantic hallucination remain critical issues; and at the decision level, it remains difficult to form a trustworthy closed loop for responsibility assessment, risk handling, and regulatory workflows.^62^ Based on this understanding, this section discusses current challenges and future directions from four perspectives: data, models, semantics, and decision-making.

### Data level: From annotated sample repositories to trajectory-database association systems

At the data level, the core bottleneck in current ship navigation behavior pattern mining is not merely the insufficient number of labeled samples, but the simultaneous limitations in openness, authenticity, semantic hierarchy, and dynamic association capability in available behavior-annotated databases.^19^^,^^32^ Existing studies commonly treat ship trajectories as isolated and statically stored training samples, with emphasis on self-constructed datasets and scale expansion. However, in open sea areas where new operational patterns and regional regulatory rules continuously evolve, the annotation of high-level ship behavior can no longer be supported by a single trajectory segment alone.^3^^,^^19^^,^^32^^,^^33^^,^^34^ Therefore, a shift is needed from manually labeled sample storage toward open, organized, and linked data.

On the one hand, underlying trajectory databases are not yet tightly linked with multi-source heterogeneous databases, such as environmental object databases and maritime knowledge bases. This limits the depth of learning and the breadth of expansion for ship navigation behavior pattern mining models. On the other hand, trajectory databases and identified behavior pattern databases have not yet formed feedback iteration, matching, and counter-evidence mechanisms. As a result, model outputs often remain one-way and difficult-to-verify probabilistic hypotheses, lacking the underlying data-flow support required for knowledge inheritance, updating, and evolution.^39^^,^^40^^,^^62^^,^^100^

In operational settings such as vessel traffic service surveillance, port-call monitoring, fisheries enforcement, and cross-agency maritime regulation, the data-level challenge is manifested in incomplete AIS reporting, heterogeneous sampling intervals, delayed updates, fragmented ownership, and weak linkage among trajectory, environmental, hydrographic, and regulatory databases. Existing systems therefore have difficulty maintaining a temporally synchronized and auditable representation of evolving ship behavior. Candidate engineering evaluation dimensions include trajectory completeness, missing-message ratio, update and ingestion latency, cross-source entity-matching accuracy, the proportion of labels with traceable provenance, database retrieval latency, and refresh frequency of environmental and regulatory information.^19^^,^^22^^,^^23^^,^^32^^,^^39^^,^^40^^,^^62^^,^^100^ These dimensions are reported or proposed across different study contexts; their definitions and acceptable thresholds are application-specific and should be stated rather than assumed.

Future work should promote the construction of trajectory-behavior databases with spatiotemporal semantic linkage, while further developing artificial intelligence for databases (AI4DB) and database-driven artificial intelligence (DB4AI) technologies.^100^ On the one hand, LLMs and knowledge graph technologies can be used to support the automatic construction and dynamic maintenance of trajectory databases, environmental object databases, maritime knowledge bases, and identified behavior pattern databases. This would help break data silos and enable dynamic expansion of behavior labels and cross-source knowledge mapping.^39^^,^^40^^,^^62^^,^^83^^,^^84^^,^^87^ On the other hand, when a mining model proposes an initial behavior hypothesis, the system should be able to trigger cross-database joint queries. For example, an environmental object database can be used to verify berth geometry, a behavior pattern database can be retrieved to compare historical temporal templates, a maritime knowledge base can be queried to check speed-limit rules, and multi-source information can be used to constrain model outputs. A spatiotemporal semantic linkage system across multi-source heterogeneous databases would support ship navigation behavior pattern mining in moving from fragile one-way black-box prediction toward a trustworthy inference paradigm based on bidirectional learning between model hypotheses and database information.^39^^,^^40^^,^^62^^,^^100^

### Model level: From sequence fitting to physical constraints and multi-agent interaction modeling

Existing ship behavior models are mostly centered on sequence fitting and representation learning. Their learning objectives usually focus on fitting historical data distributions or improving prediction accuracy, making it difficult to guarantee that model outputs conform to ship motion mechanisms and maritime traffic interaction regularities.^68^^,^^69^^,^^70^^,^^71^^,^^72^^,^^73^^,^^74^^,^^75^^,^^76^^,^^77^^,^^78^^,^^79^^,^^80^ For a given distribution of observed trajectory data, multiple function combinations may fit the spatiotemporal process. However, models that also satisfy domain priors such as ship physical motion mechanisms and navigation-rule constraints usually form only a limited subset.^43^^,^^44^^,^^45^^,^^46^^,^^47^^,^^48^^,^^49^^,^^50^^,^^51^^,^^69^ The three-layer framework proposed in this review—physical behavior, interactive behavior, and semantic behavior—provides complementary information from multiple perspectives and can serve as an important information-learning pathway for future model development. Therefore, the core challenge is how to construct collaborative learning frameworks and intelligent models that jointly satisfy low-level motion regularities, middle-level interaction regularities, and high-level behavioral regularities.

At the model level, relevant industrial scenarios include collision-avoidance support in ports and narrow waterways, multi-ship encounter prediction, autonomous navigation, and traffic-flow management under congestion. Current systems are limited by physically infeasible outputs, incomplete coverage of interacting encounter events, sensitivity to regional traffic distributions, and a gap between offline predictive accuracy and reliability under closed-loop interaction. Evaluation of behavior or prediction models may consider physical-constraint violations, uncertainty calibration, inference latency, and degradation under cross-area or cross-vessel transfer. Evaluation of collision-avoidance or navigation algorithms under interactive conditions additionally requires scenario and encounter-event coverage, collision or near-miss outcomes, minimum separation, and rule-consistency measures.^43^^,^^44^^,^^45^^,^^46^^,^^47^^,^^48^^,^^49^^,^^50^^,^^51^^,^^68^^,^^69^^,^^70^^,^^71^^,^^72^^,^^73^^,^^74^^,^^75^^,^^76^^,^^77^^,^^78^^,^^79^^,^^80^ The two evaluation targets should not be combined into a single undifferentiated metric list.

Future models should further integrate physical constraints with multi-ship interaction mechanisms. Ship speed variation, rate-of-turn limits, inertia, reachable regions, and navigable-water constraints may be incorporated into model architectures, objective functions, or post-processing procedures to improve the physical feasibility of recognized or predicted behaviors.^43^^,^^44^^,^^45^^,^^46^^,^^47^^,^^48^^,^^49^^,^^50^^,^^51^^,^^69^ Meanwhile, multi-ship interaction models should explicitly represent mutual influence, traffic-flow organization, and navigational-rule constraints in encounter, overtaking, collision-avoidance, cooperative-navigation, and congestion scenarios.^43^^,^^44^^,^^45^^,^^46^^,^^47^^,^^48^^,^^49^^,^^50^^,^^51^^,^^75^^,^^76^^,^^77^^,^^78^^,^^79^^,^^80^

Multi-ship interaction modeling should, however, be distinguished from reliability evaluation under dynamic interactive conditions. The former concerns the representation and prediction of inter-vessel dependencies, whereas the latter examines whether collision-avoidance or navigation algorithms remain robust across diverse encounter configurations and surrounding-vessel responses. Future evaluation should therefore consider scenario coverage, overlapping or sequential encounter events, confined waterways, and cooperative or non-cooperative responses from neighboring ships. Interactive testing provides a useful pathway for exposing operational limitations and failure-prone interactions in multi-ship encounter scenarios.^101^ Direction- and environment-constrained space-time-prism methods may further support scenario construction and risk validation in confined waters.^96^^,^^97^ Accordingly, predictive performance should not be used alone as evidence of operational safety.

### Semantic level: From behavior label generation to context-driven intention explanation

The semantics of ship behavior are strongly context-dependent. The same trajectory pattern may correspond to different behavioral meanings in different sea areas, ship types, task stages, and traffic relationships.^3^^,^^33^^,^^34^^,^^98^ Therefore, the core issue at the semantic level is how to explain the possible intention behind the same behavior distribution under different contexts. Existing semantic modeling methods mostly rely on static labels or rule combinations, annotating trajectory segments as behavior categories such as “staying” or “entering”, but they insufficiently characterize the contextual mechanisms through which behavioral semantics are generated.^3^^,^^33^^,^^34^^,^^39^^,^^40^ This issue becomes more prominent when LLMs participate in behavior explanation. LLMs can generate fluent and complete explanatory texts, but they may also rely on language priors or commonsense templates to interpret trajectory phenomena with insufficient evidence as definite high-level intentions, thereby producing semantic hallucination in behavior explanation.^83^^,^^84^^,^^85^^,^^86^^,^^87^^,^^88^^,^^89^^,^^90^

At the semantic level, representative applications include port anomaly monitoring, navigational-intention interpretation, illegal-entry or illegal-fishing surveillance, and explanation of rule-related behavior. Existing systems remain constrained by context-dependent ambiguity, static label systems, incomplete rule and scenario knowledge, and the possibility that generative models produce plausible but unsupported interpretations. Evaluation should be matched to the output type: event or intention inference requires context-conditioned discrimination and calibration; anomaly assessment requires a stated reference and unknown-behavior handling; rule-related interpretation requires consistency with applicable rules; and generated explanations require evidence completeness, fidelity, and traceability. Transfer across sea areas, vessel types, and operational tasks should be reported separately.^3^^,^^33^^,^^34^^,^^39^^,^^40^^,^^83^^,^^84^^,^^85^^,^^86^^,^^87^^,^^88^^,^^89^^,^^90^^,^^98^^,^^99^ No single semantic metric is sufficient for all of these tasks.

Future research at the semantic level should shift from static label learning toward context-sensitive intention explanation. On the one hand, sea-area functions, port operation procedures, ship types, historical activity patterns, and maritime knowledge graphs should be introduced as contextual priors, so that the same trajectory pattern can be interpreted differently across scenarios.^39^^,^^40^^,^^62^^,^^98^ On the other hand, mechanisms for representing semantic uncertainty should be established. For behaviors that may have multiple possible interpretations, candidate semantics, confidence levels, and applicable conditions should be provided, rather than directly outputting a single deterministic label.^87^^,^^88^^,^^89^^,^^90^ Accordingly, the objective of the semantic level should move from “assigning a behavior name” toward “explaining what the behavior means in a specific scenario”, while suppressing semantic hallucination that lacks contextual evidence.

### Decision level: From behavior recognition results to explainable regulatory evidence chains

The outputs of existing ship behavior mining methods are mostly behavior labels, anomaly scores, or predicted trajectories. These outputs can provide references for situational awareness, but a clear gap remains between such outputs and executable decisions in maritime regulation.^43^^,^^44^^,^^45^^,^^46^^,^^47^^,^^48^^,^^49^^,^^50^^,^^51^^,^^87^^,^^88^^,^^102^ Practical management tasks require not only judging what a ship has done, but also answering whether the behavior truly occurred, how responsibility should be assessed, and whether intervention is needed. A single behavior label or probability score is insufficient for high-risk decision-making tasks such as accident tracing, violation determination, and responsibility attribution. This problem becomes more pronounced when intelligent generative models are involved. A model may provide complete behavior explanations and handling suggestions, but without trajectory evidence, rule-based grounds, and uncertainty statements, its output remains only a “best guess” and cannot be directly used as a basis for regulatory responsibility attribution. In scenarios involving missing trajectories, device shutdown, abnormal messages, or complex multi-ship interactions, incorrect recognition or over-interpretation may lead to false alarms, missed detections, or even misattribution of responsibility. Therefore, the key at the future decision level is not to make model outputs sound more like expert language, but to ensure that every decision recommendation can be examined through reasoning evidence.

At the decision level, principal industrial scenarios include vessel traffic service intervention, collision-risk warning, maritime-law enforcement support, accident investigation support, and human-led responsibility analysis. Current systems commonly provide isolated labels, scores, or predicted trajectories without a complete chain linking observations, spatial calculations, applicable rules, uncertainty, and counter-evidence; their outputs are therefore difficult to audit or translate into proportionate action. Candidate engineering dimensions include false-alarm and missed-detection rates, warning lead time, decision latency, evidence-chain completeness, traceability of each recommendation to source data and rules, human-review time, intervention effectiveness, and reproducibility during post-event audit.^43^^,^^44^^,^^45^^,^^46^^,^^47^^,^^48^^,^^49^^,^^50^^,^^51^^,^^87^^,^^88^^,^^102^ These measures evaluate decision support and auditability; they do not validate automatic legal responsibility attribution, which remains dependent on competent authorities, procedural rules, and human review.

Future work needs to construct evidence-driven trustworthy behavior decision mechanisms. Beyond behavior recognition results, such mechanisms should also output trajectory evidence chains, spatial relationship calculation results, historically similar cases, rule-compliance checks, confidence intervals, and potential counter-evidence.^87^^,^^88^^,^^102^ For example, when a system gives a judgment of “suspected illegal crossing of a prohibited navigation area”, it should provide not only the behavior category, but also the corresponding trajectory segment, entry and exit times, topological relationships with the boundary of the prohibited area, applicable regulatory clauses, comparisons with historically similar behaviors, and sources of uncertainty. In this way, maritime decision-making can shift from “trusting model outputs” to “examining evidence chains”.

## Conclusions

This Review synthesizes data sources, annotated datasets, behavior representations, mining methods, applications, and research challenges in ship navigation behavior pattern mining. Its central conclusion is that kinematic patterns, vessel-object relations, context-conditioned event meanings, regional traffic aggregates, and downstream risk estimates should not be treated as equivalent analytical outputs. The proposed three-layer framework organizes physical, interactive, and semantic representations as levels of abstraction, while single-vessel, pairwise or multi-agent, and regional or collective analysis constitute an independent aggregation scale. The framework is conceptual and comparative rather than a formal ontology or an empirically validated end-to-end architecture. The four analytical groups are also non-mutually exclusive, but they are not fully symmetrical: explicit knowledge, engineered-feature statistics, and learned representations are primary inference paradigms, whereas large-model assistance is principally a cross-cutting retrieval, semantic-integration, and tool-orchestration layer. They should therefore be combined and evaluated according to target outputs, evidence availability, uncertainty, traceability, and safety consequences rather than ranked as a linear technical evolution. Future work should prioritize traceable behavior labels and linked data, physically and rule-consistent interaction models with coverage-oriented testing, context-conditioned semantic inference that preserves uncertainty and alternative explanations, and auditable links from behavior evidence to separate risk and decision models. Progress should be evaluated through clearly defined, task-specific measures of data provenance, physical and rule consistency, transfer robustness, calibration, evidence fidelity, and intervention effectiveness, without interpreting behavior labels or generated explanations as direct accident probabilities or automatic regulatory conclusions.

## Acknowledgments

This work was supported by the Innovation Science Foundation of 10.13039/501100007085National University of Defense Technology (grant no 25-ZZCX-ZX-CXXM-08).

## Author contributions

Conceptualization, Y.X., D.W., and L.C.; methodology, Y.X.; investigation, Y.X.; data curation, Y.X.; writing – original draft, Y.X.; writing – review and editing, Y.X., D.W., and L.C.; visualization, Y.X.; supervision, D.W. and L.C.; project administration, L.C.; funding acquisition, L.C.

## Declaration of interests

The authors declare that they have no financial interests.
